# Patched-Based Swin Transformer Hyperprior for Learned Image Compression

**DOI:** 10.3390/jimaging12010012

**Published:** 2025-12-26

**Authors:** Sibusiso B. Buthelezi, Jules R. Tapamo

**Affiliations:** Discipline of Electrical, Electronic and Computer Engineering, University of KwaZulu-Natal, Durban 4041, South Africa; sbjbuthelezi@gmail.com

**Keywords:** differentiable quantization module, variational inference, swin transformer hyperprior, rate-distortion optimization, latent variable modelling

## Abstract

We present a hybrid end-to-end learned image compression framework that combines a CNN-based variational autoencoder (VAE) with an efficient hierarchical Swin Transformer to address the limitations of existing entropy models in capturing global dependencies under computational constraints. Traditional VAE-based codecs typically rely on CNN-based priors with localized receptive fields, which are insufficient for modelling the complex, high-dimensional dependencies of the latent space, thereby limiting compression efficiency. While fully global transformer-based models can capture long-range dependencies, their high computational complexity makes them impractical for high-resolution image compression. To overcome this trade-off, our approach couples a CNN-based VAE with a patch-based hierarchical Swin Transformer hyperprior that employs shifted window self-attention to effectively model both local and global contextual information while maintaining computational efficiency. The proposed framework tightly integrates this expressive entropy model with an end-to-end differentiable quantization module, enabling joint optimization of the complete rate-distortion objective. By learning a more accurate probability distribution of the latent representation, the model achieves improved bitrate estimation and a more compact latent representation, resulting in enhanced compression performance. We validate our approach on the widely used Kodak, JPEG AI, and CLIC datasets, demonstrating that the proposed hybrid architecture achieves superior rate-distortion performance, delivering higher visual quality at lower bitrates compared to methods relying on simpler CNN-based entropy priors. This work demonstrates the effectiveness of integrating efficient transformer architectures into learned image compression and highlights their potential for advancing entropy modelling beyond conventional CNN-based designs.

## 1. Introduction

Learning-based compression represents a breakthrough in image and video compression by leveraging the power of deep learning, to improve both compression efficiency and perceptual quality. Traditional compression methods, such as JPEG [1], MPEG, and H.264 [2], rely on carefully designed pipelines involving stages like transform coding, quantization, and entropy coding. While these techniques are efficient and widely adopted, they struggle to optimize both bit rate and visual quality, especially at low bit rates where visual artifacts become prominent. The advent of deep learning introduced a more adaptive approach, with learning-based methods capable of dynamically adjusting to diverse image content. Instead of following rigid, hand-crafted steps, learning-based approaches utilize neural networks to automatically identify patterns, remove redundancies, and capture essential information in the data.

Pioneering works by Ballé et al. [3] established a foundational approach to learning-based compression by structuring it as an end-to-end problem, where the entire compression process is optimized through a single network. Ballé et al. introduced a deep network that compresses images by learning a non-linear transform in conjunction with quantization and entropy modelling [4]. The integration of a trainable rate-distortion loss function allows the network to balance compression rate and visual fidelity, creating a seamless optimization process that is more efficient than traditional methods.

The Variational Image Compression with a Scale Hyperprior by Ballé et al. [3,5,6] builds upon this by adding a hyperprior, a separate network that models the statistical dependencies in the latent representation. This hyperprior provides side information about the distribution of the encoded features, which improves entropy coding efficiency and further optimizes compression rates. By estimating the variance at each spatial location, the hyperprior allows the compression model to capture fine-grained information and adapt more precisely to complex textures, resulting in visually high-quality reconstructions at lower bit rates. This work is instrumental in enhancing the quality and efficiency of deep learning-based compression systems, particularly in capturing spatial dependencies in the image.

Additional advances in learning-based compression are exemplified by studies like the works presented in [7,8]. These works address the challenge of controlling bit rate during compression, a crucial factor for applications with variable bandwidth or storage constraints. Guerin et al. [7] introduces methods to constrain the rate within a target range, ensuring that the compressed output meets specific storage or transmission requirements. Meanwhile, Toderici et al. [8] employ RNNs to compress images at different bit rates within the same model, allowing flexible adaptation to bit rate requirements without retraining. The recurrent structure of RNNs enables these networks to sequentially process data, providing an efficient means of handling compression at various rates by reusing previously learned information. Collectively, these approaches push the boundaries of what learning-based image compression can achieve. By leveraging architectures that integrate latent space priors, rate control, and variable-rate capabilities, learning-based methods are proving more adaptable and efficient compared to traditional techniques. These advancements enable optimized compression pipelines that deliver high visual quality at lower bit rates, addressing both practical constraints and perceptual quality improvements [9,10].

In this work, we propose a novel framework for learned image compression that directly addresses the limitations of existing approaches by significantly improving the entropy model. Our focus is on both pure CNN-based and transformer-based models. CNN-based methods are limited by their reliance on local windowed attention, which captures only short-range dependencies and lacks global context, whereas transformer-based methods often incur prohibitive computational and training costs due to the quadratic complexity of the attention mechanism. The proposed method combines the strengths of both domains in an efficient architecture: it employs fast and lightweight CNNs for encoding and decoding, while leveraging a hierarchical Swin Transformer with shifted window attention to capture global context. This hybrid design is particularly well-suited for compression, as the windowed attention mechanism effectively models both local and long-range spatial dependencies in the latent representation, which are essential for accurate bitrate estimation.

Our framework integrates this powerful prior with a learned, end-to-end differentiable quantization module. The joint optimization of these components ensures that the entire compression pipeline from encoding to bitstream generation is optimized for both reconstruction quality and bitrate. At its core, the method performs unified rate-distortion optimization, coupling the quantization process with the Swin Transformer’s noise regularization, thereby aligning entropy estimation with perceptual fidelity. Extensive evaluations on benchmark datasets, including Kodak, JPEG AI, and CLIC datasets, demonstrate the effectiveness of our approach, achieving superior performance in terms of rate-distortion trade-offs and perceptual quality.

This research makes the following contributions:

- Expressive Entropy Model: Our Patch-based Swin Transformer Prior is a novel application of hierarchical transformers for entropy modelling, uniquely capable of capturing both local and global dependencies in the latent space with linear complexity;
- Jointly Learned Quantization: We introduce a Differentiable Learned Quantization module with an auxiliary loss, which adapts the discrete representation to the data, moving beyond static quantization and further closing the gap between training and inference;
- Co-operative Optimization: The entire framework, including encoder, decoder, entropy prior, and quantization codebook, is optimized jointly end-to-end, allowing each component to co-adapt for a globally optimal rate-distortion trade-off.

## 2. Related Work

The development of learned image compression has evolved significantly with the introduction of key concepts and models that have shaped the field. One of the foundational works is the Variational Auto-Encoding Bayes (VAE) introduced in [11]. It plays a crucial role in unsupervised learning and probabilistic models, offering an efficient probabilistic framework for encoding and decoding image distributions. This model underpins much of the current work in learned image compression, providing a probabilistic approach that helps in efficiently capturing the image data for compression purposes [3,5]. Theis et al. [12] proposed a Compressive Autoencoder for lossy image compression. Their work marked a significant step toward reducing the storage requirements for images by leveraging autoencoders. This approach facilitated a shift from traditional image compression methods like JPEG [13], paving the way for deep learning models to be integrated into image compression techniques. The introduction of autoencoders emphasized the potential for neural networks to improve compression efficiency while maintaining image quality. Another pivotal advancement came with [3], with a proposal for an End-to-End Optimized Image Compression framework. This model introduced the concept of jointly training both the encoder and decoder in an end-to-end fashion, optimizing the entire compression pipeline for superior performance. Their approach surpassed traditional techniques such as JPEG by utilizing deep learning methods, setting the stage for more sophisticated learned image compression models in subsequent research.

Building on this, [5] introduced a scale hyperprior in their model to improve entropy modelling. This new approach enhanced the efficiency of the compression process by more effectively modelling the prior distribution of image data, resulting in better compression performance. The scale hyperprior provided a more robust method for capturing image details, leading to improved compression ratios and image quality. Further advancements were made in [14] by combining autoregressive models with hierarchical priors to improve compression quality. The approach used in this work enhanced the fidelity of reconstructed images by incorporating autoregressive models that better modelled the dependencies between pixels and hierarchical priors that efficiently captured long-range correlations in the image data. This development marked a significant step forward in the modelling of image priors, leading to a more efficient and accurate compression process.

The refinement of entropy modelling and prior techniques played a crucial role in enhancing learned image compression models. In [15], the introduction of image-dependent local entropy models represented a significant advancement in the field. This approach tailored the entropy model based on the specific content of each image, allowing for a more adaptive and efficient compression process. By leveraging image-specific information, their model improved compression efficiency and image quality, representing a notable leap forward in how image content could be integrated into the compression pipeline. The work in [6] focused on improving the computational efficiency of neural image compression. It developed methods to reduce the processing cost while maintaining high compression performance. This addressed a key limitation of early learned compression models, which often struggled with computational overhead. Johnston et al.’s contributions made learned compression more practical for real-world applications, where computational resources are often limited. Further extending the work on priors, ref. [16] introduced a coarse-to-fine hyper-prior modelling approach for learned image compression. The  hierarchical method proposed progressively refined the prior modelling process, improving both flexibility and performance. This coarse-to-fine approach allowed for a more nuanced understanding of image features, enabling higher compression quality while reducing redundancy. It built upon the earlier work presented in [5], offering a more sophisticated approach to prior modelling that enhanced compression efficiency.

As learned image compression models evolved, a key focus shifted toward improving their computational efficiency which led to the authors in [5] proposing the use of octave convolutions for learned multi-frequency image compression. By reducing spatial redundancy and better capturing high-frequency image details, octave convolutions allowed for more efficient compression across different image frequencies. This approach marked a significant improvement in how neural networks handle multi-frequency information, leading to more compact and accurate representations of image data. Building upon octave convolutions, ref. [17] introduced variable-rate multi-frequency image compression using modulated generalized octave convolution. This work provided a flexible framework for handling varying image frequencies more effectively, enabling compression systems to adapt to different levels of detail and visual content. This innovation contributed to more adaptive compression strategies, where the model can vary the compression rate based on the specific characteristics of the image.

Recent advancements in learned image compression have introduced novel architectures and models that further enhance compression efficiency and image quality. Ref. [18] proposes a fully transformer-based compression pipeline that leverages the self-attention mechanism throughout the entire autoencoder structure, including both the analysis, synthesis transforms and the hyperprior model. By designing the entire system, including the crucial context model, using only attention layers, this paper seeks to maximize the benefits of global dependency modelling at every stage of the codec. This comprehensive application of the Transformer aims to push the theoretical boundaries of Rate-Distortion (R-D) performance by achieving superior decorrelation and highly accurate statistical modelling of the latent features. This end-to-end attention approach establishes the feasibility of a high-performance codec relying exclusively on the powerful, but computationally demanding, transformer architecture.

In another significant development [19] presents a compression framework that is purely attention-based for the analysis and synthesis transforms but distinctively anchors its statistical modelling using the highly robust and established Ballé variational hyperprior [5]. By focusing the innovation on the full transformer encoder, decoder while utilizing a proven CNN-based hyperprior, the study effectively isolates the performance gain attributable solely to the Transformer’s superior feature modelling capabilities. This design choice proves the architectural sufficiency of attention for the transform stages while ensuring high entropy coding accuracy, offering a powerful, yet practical, alternative to fully convolutional codecs.

Further advancements in the field represents the MambaVC [20] a landmark effort in applying the Selective State Space (Mamba) model specifically to visual compression. The core contribution is the development of the 2D Selective Scanning (2DSS) module, which ingeniously adapts the inherently one-dimensional processing of Mamba to the two-dimensional nature of images. This architecture achieves competitive compression performance while simultaneously offering vastly superior scalability and efficiency compared to both transformer and classic CNN-based methods. MambaVC effectively establishes the Mamba architecture as a powerful, efficient, and computationally scalable alternative to attention for learned image compression, focusing on high practicality without compromising quality.

Extending from this MambaVC [20], ref. [21] addresses the computational and scalability drawbacks associated with transformer-based methods by introducing an architecture based on Advanced State Space Models (SSMs). SSMs, particularly variants related to the Mamba architecture, offer a mechanism to model long-range dependencies with a linear computational complexity relative to the sequence length, significantly improving throughput and reducing memory footprint compared to the quadratic cost of self-attention. By replacing or augmenting attention blocks with SSM layers, this approach achieves a strong balance between R-D performance and computational efficiency, making the resulting model a highly practical candidate for high-resolution, resource-constrained applications.

Collectively, these approaches continue to push the boundaries of learned image compression. While they highlight the representational strengths of diverse neural network architectures, a persistent challenge remains: designing an entropy model that is both highly expressive and computationally efficient. Simple priors offer superior computational speed but lack the capacity to capture the complex, non-local statistical dependencies within the latent feature distributions. In contrast, powerful autoregressive or fully attention-based context models are often too slow for practical, high-throughput deployment due to high latency. Recent progress in pure transformer architectures suggests new possibilities, but these models inherently suffer from the quadratic scaling issue ($O(N^{2})$) of standard self-attention, which severely limits their efficiency and scalability for high-resolution images.

Our work directly addresses this critical gap by introducing a Swin Transformer-based entropy model that integrates a powerful, yet locally constrained, attention mechanism within a hierarchical framework. This enables the creation of an expressive yet efficient prior model. The overall system employs a CNN-based encoder decoder for fast and efficient feature transformation (analysis and synthesis), while leveraging the Swin Transformer-based entropy model to accurately capture long-range dependencies and global context within the latent space. By strategically combining the complementary strengths of both architectures CNNs for translational invariance and computational efficiency in the spatial transforms, and transformers for representational power and global context modelling in the statistical prior our hybrid model establishes a new, highly efficient benchmark in learned image compression.

## 3. Materials and Methods

Learned image compression, pioneered by works such as Ballé et al. [3,5], has established a robust framework for achieving a superior trade-off between compression rate and reconstruction quality. These methods operate by training an end-to-end codec, which consists of an encoder that maps an image *x* to a compact latent representation *y*, and a decoder that reconstructs the image $\hat{x}$ from *y*. The entire system is optimized to minimize a rate-distortion (R-D) loss function, which is a weighted sum of the distortion (reconstruction error) and the estimated bitrate (rate).

The core of this probabilistic framework lies in its ability to model the latent distribution. The rate term is typically derived from the entropy of the latent representation, which is estimated by approximating the posterior distribution p(y∣x) with a tractable prior p(y) and a hyperprior p(z) that captures the dependencies in the latent space. The objective is to minimize a combined loss:

(1) $$L=D\left(x,\hat{x}\right)+\lambda R\left(y\right)$$

where *D* is the distortion, *R* is the rate, and λ is a hyperparameter balancing the two. The rate term, derived from the negative log-likelihood of the quantized latent variables, is crucial for effective compression.

A significant contribution of foundational work was the introduction of the scale hyperprior, which uses a separate network to model the statistical dependencies in the latent representation. This marked a key advancement over simple Gaussian priors by allowing for adaptive entropy coding. However, as evident by the architectural design, these hyperpriors, often based on convolutional networks, possess inherent limitations. They struggle to effectively capture the intricate, long-range dependencies that exist across a large latent space. While a convolutional network’s receptive field can grow with depth, it remains fundamentally local, limiting its ability to model global context. This often leads to sub-optimal rate estimation and, consequently, reduced compression efficiency.

The use of traditional autoregressive models, which model each latent variable based on its neighbours, offers a more powerful solution. Yet, they are fundamentally sequential, meaning they are computationally expensive and slow to decode. This makes them impractical for real-time applications where rapid decompression is required. Therefore, a critical challenge remains developing a prior that is expressive enough to capture complex dependencies for optimal compression yet efficient enough for practical implementation.

### 3.1. Variational Inference Model

In this research, a variational inference model [3,5] that approximates the true posterior p(y|x) with a simpler distribution q(y|x). The goal is to make q(y|x) as close as possible to p(y|x) by minimizing Kullback-Leibler (KL) divergence, computed over the data distribution p(x). The model is trained by minimizing the following expected loss, adapted from:

(2) $$\begin{array}{cc} E_{x∼p_{x}}D_{KL}\left[p∥p_{\tilde{y},\tilde{z}|x}\right]=E_{x∼p_{x}}E_{\tilde{y},\tilde{z}∼q} & [\log q\left(\tilde{y},\tilde{z}|x\right)-\log p_{x|\tilde{y}}\left(x|\tilde{y}\right)\\ \; & -\log p_{\tilde{y}|\tilde{z}}\left(\tilde{y}|\tilde{z}\right)-\log p_{\tilde{z}}\left(\tilde{z}\right)] \end{array}$$

The expectation over $x∼p_{x}$ ensures the loss considers all possible input data. The KL divergence measures how closely the model’s approximate posterior $q(\tilde{y},\tilde{z}|x)$ matches the true joint posterior p(y,z|x), with smaller divergence indicating better approximation. The first term, $\log q(\tilde{y},\tilde{z}|x)$, comes from the encoder’s approximate posterior, which maps the input *x* to latent variables $\tilde{y}$ and $\tilde{z}$. It represents the inference process in the variational model and is part of the evidence lower bound (ELBO). The second term $-\log p_{x|\tilde{y}}\left(x|\tilde{y}\right)$ expresses the reconstruction likelihood, evaluates the likelihood of reconstructing *x* from $\tilde{y}$. It reflects the distortion component in the rate-distortion trade-off. The third term $\log p_{\tilde{y}|\tilde{z}}\left(\tilde{y}|\tilde{z}\right)$ expresses the conditional prior, captures the hierarchical relationship between $\tilde{y}$ and $\tilde{z}$, ensuring $\tilde{y}$ aligns with the prior defined by $\tilde{z}$. The prior $p_{\tilde{y}|\tilde{z}}\left(\tilde{y}|\tilde{z},\theta_{h}\right)$ is designed to combine the properties of Gaussian and uniform distributions, enabling the model to effectively capture both global patterns and local variations in structured data [3,5], expressed as:

(3) $$\begin{array}{cc} p_{\tilde{y}|\tilde{z}}\left(\tilde{y}|\tilde{z},\theta_{h}\right) & =\prod_{i}\left[N\left(0,{\tilde{\sigma }}_{i}^{2}\right)*U\left(-\frac{1}{2},\frac{1}{2}\right)\right]\left({\tilde{y}}_{i}\right),\\ \tilde{\sigma } & =h_{s}\left(\tilde{z};\phi_{h}\right) \end{array}$$

where the Gaussian component, $N(0,{\tilde{\sigma }}_{i}^{2})$, represents uncertainty around each latent variable $y_{i}$, with a mean of zero and variance ${\tilde{\sigma }}_{i}^{2}$. This variance is not fixed but adaptively generated as $\tilde{\sigma }=h_{s}\left(\tilde{z};\phi_{h}\right)$, where $\tilde{z}$ serves as a higher-level latent representation. The fourth term $-\log p_{\tilde{z}}\left(\tilde{z}\right)$ defines the hyperprior regularization, enforces $\tilde{z}$ to conform to a predefined distribution, reducing redundancy while maintaining sufficient information for efficient representation of $\tilde{y}$.

### 3.2. Proposed Model

We propose a novel end-to-end learned image compression framework that fundamentally advances entropy modelling. Our core contribution is a Patch-based Swin Transformer Prior that captures both local and long-range dependencies in the latent space [22,23,24], coupled with a Differentiable Learned Quantisation module that adapts its codebook to the data distribution [25,26]. This joint effort achieves a superior rate-distortion trade-off.

**Figure 1:**
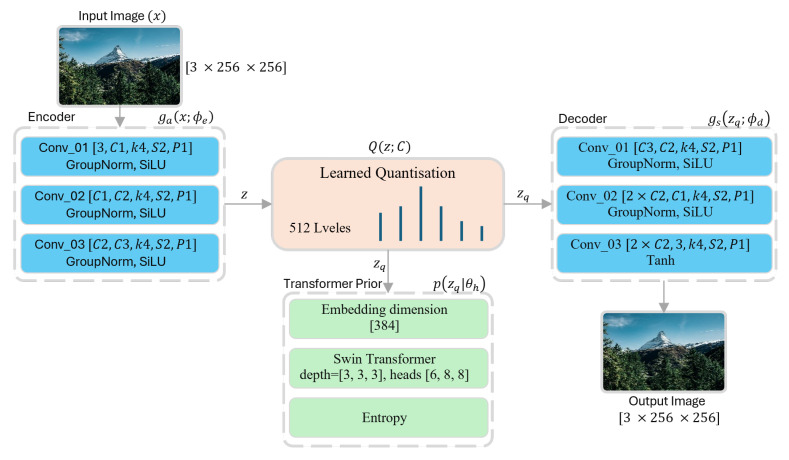
A variational autoencoder (VAE) incorporating a Transformer-based hyperprior with a Swin Transformer attention mechanism is employed to preserve both local and global features. The latent tensor produced after the quantization module is partitioned into 4×4×ch patches, each of size 8×8×ch. These patches are subsequently tokenized to form a total of 16 tokens, which are then linearly embedded into a 384-dimensional feature space. The embedded tokens are processed by a stack of three Swin Transformer blocks with multi-head self-attention configurations of [6,8,8] heads, respectively. The resulting output is passed through a decoder network that maps the transformer features to the parameters of the entropy model, producing mean and scale tensors used for entropy coding.

Our model operates as a variational autoencoder as illustrated in Figure 1 where an input image, *x*, is first transformed into a continuous latent representation, *z*, through an encoder, $g_{a}\left(x;\phi_{e}\right)$. The continuous latent vector *z* is then passed to the quantization module Q(z;C). This module maps the continuous values of *z* to a set of discrete symbols $z_{q}$ from a codebook *C*. This step is crucial for compression, as discrete symbols can be efficiently encoded using lossless entropy coding techniques for storage or transmission. Lastly, a decoder transformation, $g_{s}\left(z_{q};\phi_{d}\right)$, reconstructs the compressed data into the output image, $\hat{x}$. The entire system is trained end-to-end to minimize the Rate-Distortion Lagrangian, which balances the compression rate and reconstruction quality:

(4) $$L=E[D\left(x,\hat{x}\right)+\lambda R\left(\hat{z}\right)]$$

Here, $D(x,\hat{x})$ is the distortion term, $R(\hat{z})$ is the compression rate, and λ is the Lagrangian multiplier that controls the rate-distortion trade-off.

Since the input image is divided into patches and processed independently, the reconstructed image is initially obtained as individual patches of size 256×256×3. To form the full image, a stitching operation is performed, where patches are overlapped by 16 pixels along both vertical and horizontal dimensions. This overlapping helps to reduce boundary artifacts and ensures smoother transitions between adjacent patches. Additionally, overlapping patches allow the model to leverage contextual information from neighboring regions, which improves reconstruction quality by mitigating discontinuities at patch edges. The final full-resolution image is obtained by blending overlapping regions, typically using averaging, to preserve visual consistency and high-frequency details.

#### 3.2.1. Swin Transformer Entropy Model

**Figure 2:**
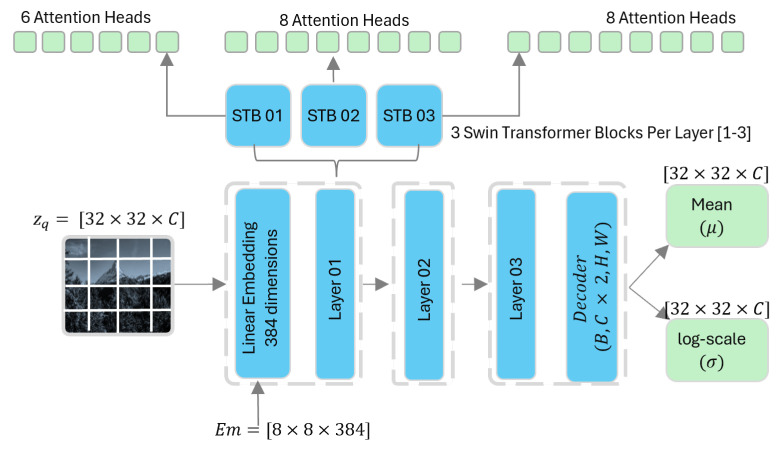
The encoder maps each input image to a fixed latent representation of size 32×32×Ch, where the channel dimension Ch controls the rate–distortion trade-off of the model. A larger number of channels improves reconstruction quality at the cost of higher bitrate, while fewer channels promote stronger compression with reduced reconstruction fidelity. The resulting latent tensor is then quantized and partitioned into smaller patches of size 8×8×Ch. This patching produces a 4×4 grid of tokens, each corresponding to a flattened vector of length 8×8×Ch. For Ch=256, each token has a dimensionality of 16,384. To reduce model complexity and computational cost, each token is linearly projected into a 384-dimensional embedding space. The embedded token sequence is subsequently processed by a stack of Swin Transformer blocks, where attention is computed to model local and global dependencies. Finally, the transformer output is decoded to predict the mean and scale parameters required for efficient entropy coding.

The core of our innovation lies in the entropy model, $p(z_{q}|\theta_{h})$, conceptually illustrated in Figure 2. This model, which predicts the parameters of a conditional Gaussian distribution, is built using a Swin Transformer. This model starts by taking the quantized latent tensor, $z_{q}\in R^{Ch\times H^{\prime }\times W^{\prime }}$, of size [32×32×Ch] and partitioning it into a sequence of patches of [8×8×Ch]. This partitioning results in a spatial token grid of (32/8)×(32/8)=[4×4] tokens, for a total sequence length of L=16.

These patches form a token space 4×4×Ch, these are subsequently projected linearly through 384 dimensions and combined with learnable positional embeddings, $E_{\mathrm{pos}}$, to create an initial sequence of tokens, $T^{\left(0\right)}$, as expressed below:

(5) $$T^{\left(0\right)}={\left[p_{1}W_{e},\ldots ,p_{N}W_{e}\right]}^{T}+E_{\mathrm{pos}},\;W_{e}\in R^{(Chp^{2})\times D}$$

where $p_{i}$ denotes the *i*th input patch from a sequence of total length *N*; $W_{e}\in R^{(Chp^{2})\times D}$ is the embedding projection matrix, which linearly maps each flattened patch vector into a model-compatible dimension *D*. Here, Ch is the number of input channels and *p* is the spatial patch size. The term ${\left[p_{1}W_{e},\ldots ,p_{N}W_{e}\right]}^{T}$ constitutes the semantic embedding matrix, formed by stacking all projected token embeddings.

The sequence of tokens are then processed by a series of Swin Transformer blocks [23]. A key feature of these blocks is the shifted window multi-head self-attention, which efficiently captures global context across the entire latent space. For a given window of tokens *X*, attention is calculated as:

(6) $$\mathrm{Attention}\left(Q,K,V\right)=\mathrm{softmax}\left(\frac{QK^{T}}{\sqrt{d_{k}}}+B\right)V$$

where $Q=XW^{Q}$, $K=XW^{K}$, $V=XW^{V}$, and *B* is a learnable relative positional bias. By shifting the windows in alternating layers, the model builds a powerful hierarchical representation of dependencies across the latent space. After passing through all layers, the final output tokens $T^{\left(L\right)}$ are reshaped and processed by a small convolutional decoder, $f_{\mathrm{out}}$, to predict the parameters mean (μ) and standard deviation (σ) for the Gaussian distribution, as follows:

(7) $$\mu ,\sigma =f_{\mathrm{out}}\left(T^{\left(L\right)};\phi_{\mathrm{out}}\right)$$

The probability for each quantized latent element, $z_{q,i}$, is then computed using the cumulative distribution function, Φ(·), of the predicted Gaussian. This allows us to calculate the total rate for the latent tensor as the negative sum of the log probabilities of its elements, as:

(8) $$P\left(z_{q,i}\right)=\Phi \left(\frac{z_{q,i}-\mu_{i}}{\sigma_{i}}\right)-\Phi \left(\frac{z_{q,i}-\mu_{i}}{\sigma_{i}}\right)$$

The total rate for the latent tensor is the negative sum of the log probabilities of its elements:

(9) $$R\left(z_{q}\right)=-\sum_{i}\log_{2}P\left(z_{q,i}\right)$$

#### 3.2.2. Differentiable Learned Quantization

Our framework also introduces a differentiable learned quantization scheme that adapts its codebook, $C=\{l_{1},l_{2},\ldots ,l_{K}\}$, to the data distribution [25]. For a continuous latent value *z*, the quantization operation finds the closest level, $l_{k^{*}}$, in the learned codebook:

(10) $$Q\left(z\right)=l_{k^{*}},\;\mathrm{where}\;k^{*}=\arg\min_{k}{∥z-l_{k}∥}_{2}^{2}$$

To allow for end-to-end training, we use a straight-through estimator (STE) during the forward pass, defined as:

(11) $$z_{q}=\mathrm{STE}\left(z,Q\left(z\right)\right)=Q\left(z\right)+\mathrm{sg}\left(z-Q\left(z\right)\right)$$

where sg(·) is the stop-gradient operator. We also introduce a new auxiliary quantization loss to directly minimize the quantization error and guide the learning of the codebook, defined as:

(12) $$L_{q}={∥z-Q\left(z\right)∥}_{2}^{2}$$

The motivation for the proposed model configuration is derived from an ablation study that analyses the impact of key architectural components on both compression performance and computational complexity. We study the effect of encoder depth by varying the number of convolutional layers in the VAE encoder. Deeper encoders improve feature extraction and reconstruction quality by expanding the receptive field but incur higher computational cost and diminishing rate–distortion gains. Our results show that three convolutional layers provide an effective balance between reconstruction fidelity and efficiency, and this configuration is used in the final model. We analysed the impact of the learned quantization module by varying the number of quantization levels. Increasing the number of levels yields finer latent representations and improved reconstruction quality, at the cost of higher bitrate and decoding complexity. Fewer levels enable stronger compression but degrade reconstruction. An intermediate number of quantization levels achieves the best rate–distortion trade-off and is therefore adopted.

We evaluate the hierarchical shifted-window Swin Transformer prior by varying its depth and attention capacity. Greater transformer capacity improves global dependency modelling and entropy estimation, enhancing compression efficiency, but significantly increases computational cost. The hierarchical windowed attention consistently outperforms CNN-based priors while avoiding the quadratic complexity of full global attention, offering a favourable performance–efficiency trade-off. These ablation results highlight the tight coupling between encoder depth, quantization resolution, and transformer capacity. Increasing any component improves performance but raises computational cost. The final configuration balances these factors to achieve high reconstruction quality at low bitrates with practical computational efficiency.

## 4. Experimental Results and Discussion

### 4.1. Experimental Setup

The model was trained for 100 epochs on 1100 images from the CLIC Professional dataset [27,28], with 1000 images used for training and 100 images reserved for validation. To ensure consistent input dimensions, all images were processed to a fixed patch size of 256×256×3: larger images were centrally cropped, and smaller images were padded. The Adam optimizer was used with an initial learning rate of $10^{-4}$, which was decayed by a factor of 0.5 every 20 epochs. Training was performed with a batch size of 16. To explore a wide range of the rate-distortion trade-off, a set of eight models was configured by varying the key loss function weight (λ) and the number of output channels in the convolutional layers, as detailed in Table 1.

To ensure a comprehensive evaluation of generalization capability and coding efficiency, all eight trained model variants were assessed on the Kodak, JPEG-AI, and CLIC benchmark datasets [27,28,29,30]. Performance was quantified using standard metrics: Bits-Per-Pixel (BPP) for rate, alongside PSNR, SSIM, and MS-SSIM for distortion. Computational efficiency was also evaluated. To provide a complete comparative analysis, the proposed model was benchmarked against established methods from two distinct architectural domains: a CNN-based variational autoencoder (VAE) domain and a transformer-based domain. Within the CNN domain, benchmarks included the Hyperprior [5] and Mean-Scale Hyperprior [15] models. In the transformer domain, comparisons were made against the SwinT-Hyperprior and SwinT-Charm architectures [18]. This cross-domain benchmarking enables a thorough assessment of the proposed model’s performance relative to state-of-the-art approaches.

### 4.2. Results for the CNN-Based Codec

This section presents a comprehensive evaluation of the proposed image compression model against three state-of-the-art baselines. The primary objective of this evaluation is to quantify the performance gains in coding efficiency (bitrate) and reconstruction quality achieved by our proposed architecture. The models were evaluated across a wide range of eight operating points, each corresponding to a different trade-off between rate and distortion. The outcomes obtained at each selected evaluation point are presented in Table 2, Table 3, Table 4 and Table 5, reporting the BPP, PSNR, SSIM, and MS-SSIM metrics across the two baseline methods as well as the proposed approach.

The proposed model exhibits superior compression efficiency, attaining the lowest bitrate (BPP) for all the chosen operating point of the R-D curve. Notably, it achieves the overall minimum BPP in the dataset 0.0796 firming its leading performance as presented in Table 2:

**Table 2:** The tabulated results present the average bit rate performance of the two baseline models and the proposed approach across eight rate–distortion trade-off points, where each value represents the mean computed over the entire JPEG AI dataset.

Model	01	02	03	04	05	06	07	08
Hyperprior	0.1630	0.2580	0.3940	0.5790	0.8010	1.1740	1.5810	2.0410
Mean-Scale Hyperprior	0.1120	0.2090	0.3670	0.5680	0.8310	1.1650	1.5630	2.0340
Proposed	0.0796	0.2360	0.4170	0.5470	0.8180	1.0500	1.5250	2.0430

Furthermore, the proposed model excels in reconstruction quality, consistently achieving the highest PSNR across all evaluated bitrates. It delivers a strong starting performance at 27.29 dB and reaches a peak of 40.35 dB, clearly surpassing all baseline models throughout the entire bitrate spectrum. This is presented as the combination of Table 2 and Table 3:

**Table 3:** Tables report the average PSNR performance of the two baseline models and the proposed method across eight rate–distortion trade-off points, with each entry corresponding to the mean value calculated over the complete JPEG AI dataset.

Model	01	02	03	04	05	06	07	08
Hyperprior	26.2310	27.9620	29.6560	31.3800	32.9210	35.4090	37.0150	38.5380
Mean-Scale Hyperprior	25.3970	27.4280	29.7430	31.6190	33.6190	35.4780	36.7120	37.6120
Proposed	27.2854	30.1020	32.6610	34.3060	36.8380	38.0970	39.6090	40.3534

In measures of perceptual and multi-scale structural similarity (SSIM and MS-SSIM), the proposed model exhibits a dominant and consistent lead. It achieves the highest SSIM across nearly the entire rate-distortion curve, peaking at 0.9737. Its performance in MS-SSIM is even more commanding, reaching up to 0.9981 and solidifying its state-of-the-art capability in preserving the visual quality most aligned with human judgment. The results of both metrics are presented in Table 4 and Table 5, respectively:

**Table 4:** The tables present the average SSIM performance of the two baseline models and the proposed method across eight rate–distortion trade-off points, where each entry denotes the mean value computed over the entire JPEG AI dataset.

Model	01	02	03	04	05	06	07	08
Hyperprior	0.7515	0.8133	0.8598	0.8957	0.9243	0.9481	0.9625	0.9732
Mean-Scale Hyperprior	0.7416	0.8101	0.8631	0.9006	0.9290	0.9499	0.9642	0.9738
Proposed	0.7902	0.8634	0.8976	0.9175	0.9530	0.9620	0.9730	0.9737

**Table 5:** The tables present the average MS-SSIM performance of the two baseline models and the proposed method across eight rate–distortion trade-off points, with each entry representing the mean value computed over the complete JPEG AI dataset.

Model	01	02	03	04	05	06	07	08
Hyperprior	0.9249	0.9481	0.9654	0.9763	0.9843	0.9898	0.9930	0.9953
Mean-Scale Hyperprior	0.9221	0.9491	0.9665	0.9778	0.9852	0.9903	0.9935	0.9956
Proposed	0.9620	0.9754	0.9840	0.9892	0.9960	0.9970	0.9975	0.9981

#### 4.2.1. Peak Signal-to-Noise Ratio (PSNR)

**Figure 3:**
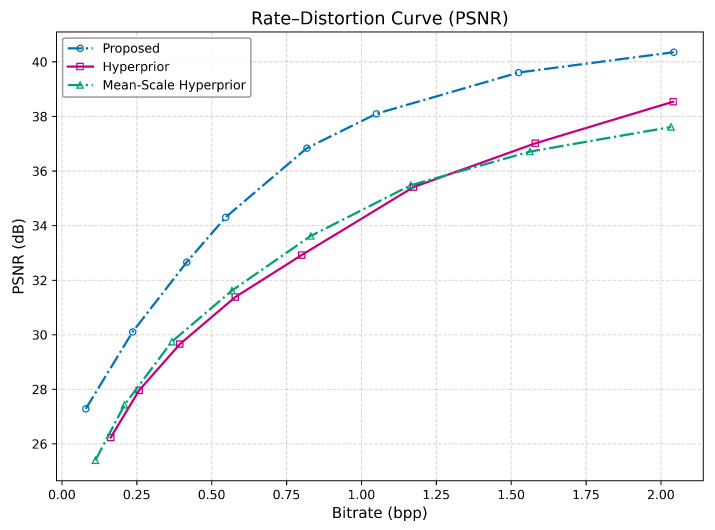
Rate-PSNR curves of the two baseline models and the proposed method across eight rate-distortion trade-off points on the JPEG AI dataset.

The proposed method establishes a superior rate-distortion performance, achieving a low bitrate of 0.0796 BPP. At this operating point, the proposed method achieves a PSNR of 27.285 dB, which is not only higher than the Hyperprior’s 26.231 dB but is also achieved at a significantly lower bitrate (0.163 BPP for Hyperprior). This trend continues into high bitrates; at approximately 1.05 BPP, the proposed method reaches 38.097 dB, a value the Hyperprior model only attains at a 70% higher bitrate of approximately 1.78 BPP. This is evident in both Table 2 and Table 3 and observable in Figure 3.

The superior PSNR indicates that the proposed method is exceptionally effective at minimizing mean-squared error. This suggests that the architecture, likely through more advanced analysis/synthesis transforms and a more powerful entropy model, preserves fine-grained details and avoids smoothing artifacts better than the baselines.

#### 4.2.2. Structural Similarity Index (SSIM)

**Figure 4:**
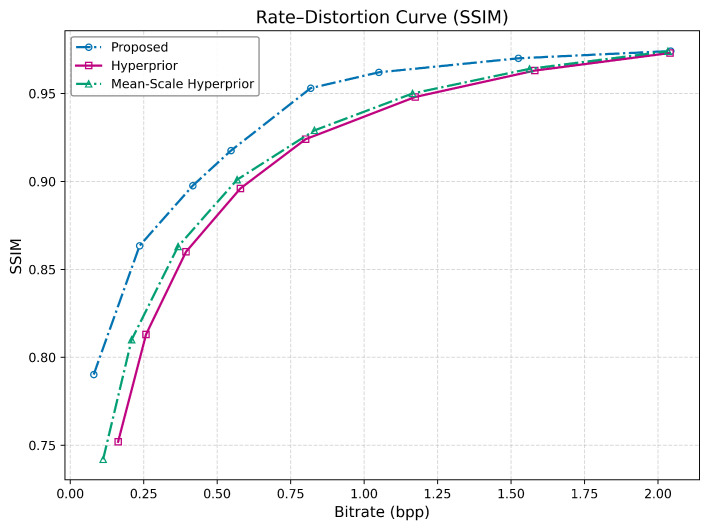
Rate–SSIM curves of the two baseline models and the proposed method across eight rate–distortion trade-off points on the JPEG AI dataset.

The proposed method demonstrates a significant advantage, particularly at lower bitrates. Its starting SSIM value of 0.790 at 0.0796 BPP is higher than what the Mean-Scale Hyperprior model achieves at 0.112 BPP. This initial lead is maintained throughout the rate-distortion curve. While the gap narrows at very high bitrates where all methods converge above 0.97 SSIM at approximately 2.0 BPP, the proposed method consistently maintains superior performance. These results are presented in Table 4 and supported by Figure 4.

The high SSIM scores confirm that the proposed method excels at preserving the structural integrity of images. The significant lead at low bitrates implies it is far more robust against common compression artifacts like blurring and blocking, which disproportionately degrade structural information.

#### 4.2.3. Multi-Scale Structural Similarity Index (MS-SSIM)

**Figure 5:**
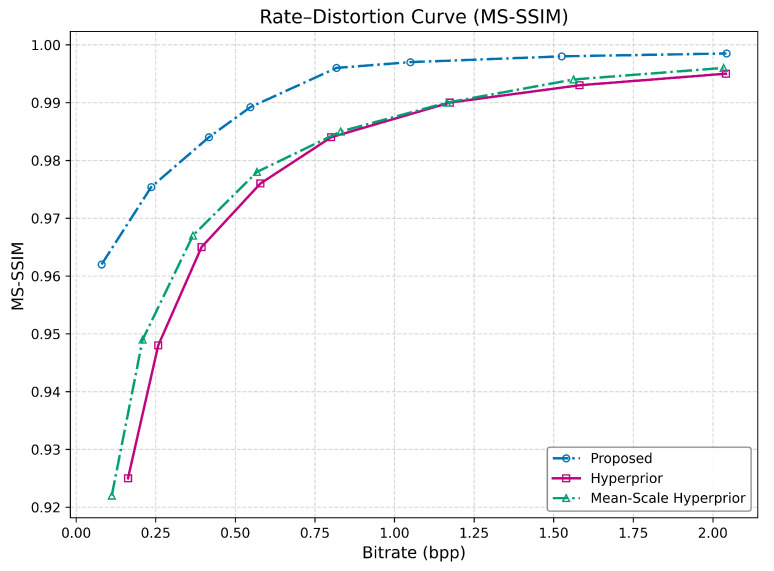
Rate-MS-SSIM curves of the two baseline models and the proposed method across eight rate-distortion trade-off points on the JPEG AI dataset.

The MS-SSIM results demonstrate the most impressive performance for the proposed method, as seen from Figure 5. The performance gap is not just significant; it is monumental. The proposed method’s MS-SSIM values are in a different league altogether, starting at an astonishing 0.962 a value that the best baseline (Hyperprior) does not reach until approximately 0.58 BPP. The proposed method achieves near-perfect reconstruction (0.996) at around 1.05 BPP, a level of quality the other methods do not approach even at their highest tested bitrates.

The exceptional MS-SSIM performance is the strongest evidence that the proposed method is highly perceptually optimized. By effectively preserving information across multiple scales (from fine edges to broader textures), it delivers a visual quality that is vastly superior to the baselines for any given bitrate. This suggests the method’s architecture is exceptionally good at prioritizing perceptually critical information during the compression process.

### 4.3. Results for the Transformer-Based Codec

For benchmarking within the transformer domain, comparisons were made against two representative architectures: SwinT-Hyperprior, featuring a full transformer-based encoder/decoder with a CNN prior; and SwinT-Charm, a fully transformer-based model with transformer encoder, decoder, and prior. Evaluation was conducted across five distinct operating points along the rate-distortion curve. The results presented are averaged across these five points and over all three benchmark datasets: Kodak, JPEG-AI, and CLIC. Beyond the core rate-distortion metrics (BPP and PSNR) used for CNN-based models, this analysis is extended to incorporate measures of computational complexity and training cost. This holistic approach provides a clear assessment of each model’s performance, evaluating not only its compression efficiency but also its practical feasibility for deployment and development. Table 6 presents the performance of the baseline models alongside the proposed method.

The most defining factor across all benchmarks is the fixed computational cost dictated by the model’s architecture. The two transformer-heavy models, SwinT-Charm and SwinT-Hyperprior, consistently required training times that were 120 to 168 times longer than the proposed method, despite utilizing approximately 1.9× more powerful (V100-class GPU) hardware. This massive overhead is a direct consequence of running the computationally expensive transformer attention mechanism within the deep VAE transforms, confirming the impracticality of the pure attention paradigm for high-throughput image compression.

When comparing the pure attention models against the proposed method from a compression standpoint, Table 7 reveals distinct performance patterns. The SwinT-Hyperprior model demonstrates significant compression inefficiency on the Kodak and CLIC datasets, requiring 35.64% and 27.39% larger file sizes, respectively, compared to the proposed model. In contrast, the SwinT-Charm model shows a markedly smaller performance gap on the JPEG-AI dataset, with only a 2.74% file size increase (though this increases to 7.51% for SwinT-Hyperprior on the same dataset). This suggests that while the proposed model maintains a decisive advantage over the SwinT-Hyperprior architecture, its lead over the fully transformer-based SwinT-Charm architecture is substantially narrower, highlighting important differences in how these competing architectures generalize across different types of image data.

The superior compression performance of the proposed method arises from the use of a powerful yet efficient transformer-based prior combined with a learned quantization model. The quantization model is jointly optimized with the full network and adapts its codebook to the characteristics of the training data, in contrast to approaches that rely on a fixed codebook.

When assessing the PSNR performance, presented in Table 8, the proposed method demonstrates a clear, dataset-dependent trade-off between exceptional practical efficiency and peak reconstruction quality. On the Kodak and JPEG-AI datasets, it sacrifices a negligible amount of PSNR a deficit of only −0.28 to −0.80 dB while achieving significantly better compression (over 20% smaller files) and using 99% less training time and 50% fewer computational resources. This makes its performance highly favourable for these image types. However, on the CLIC dataset, this trade-off shifts: the proposed method incurs a more substantial PSNR deficit of −2.4 dB (approximately 6.5% dominance), indicating its compression method is less optimal for this specific high-quality content. From a broader perspective, the proposed method positions itself as a pragmatic, efficiency-first approach, delivering near state-of-the-art quality on two major benchmarks while achieving substantial improvements in speed and computational cost. Additionally, it offers a high-compression mode for CLIC, prioritizing resource savings over maximum fidelity.

**Figure 6:**
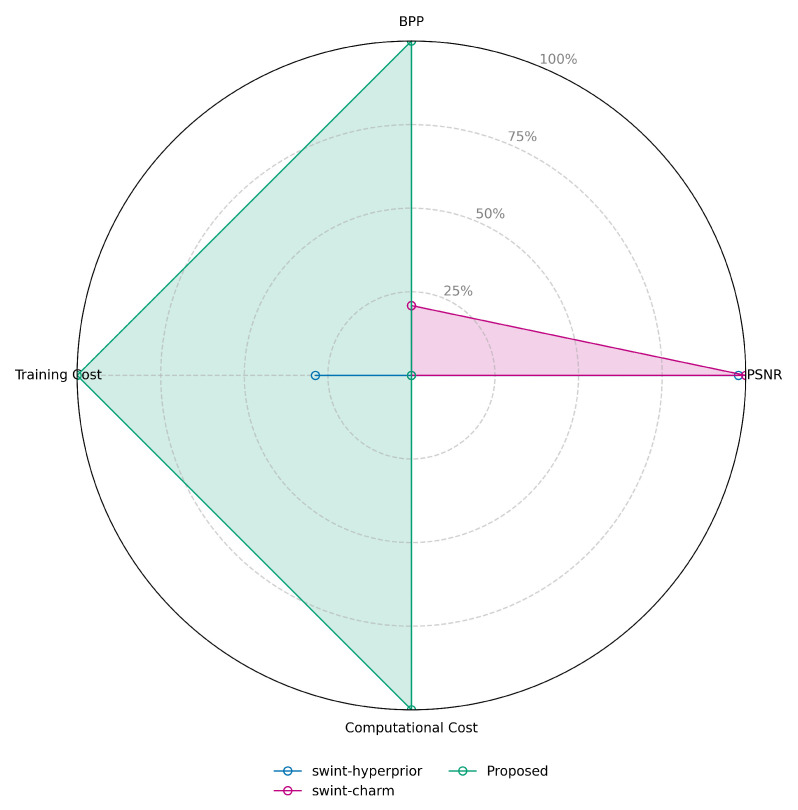
Radar plot illustrating the performance trade-offs between the swint-hyperprior, swint-charm and proposed architectures across four key metrics: BPP, PSNR, Computational Cost, and Training Cost. The swint-hyperprior model achieves maximal reconstruction fidelity, while the proposed demonstrates superior efficiency by minimizing BPP, Computational Cost, and Training Cost.

While Table 7 and Table 8 offer a numerical summary of the performance of the two baseline models and the proposed method, the dominance of the proposed model is not immediately apparent. Therefore, a radar chart is generated to clearly visualize the superior performance of the proposed method against the baseline models, as demonstrated in Figure 6.

The plot in Figure 6 normalizes all values between 0 (worst) and 1 (best) across the Kodak, JPEG AI, and CLIC datasets, inverting the scale for BPP, computational cost, and training cost (where lower values are better) while keeping the PSNR metrics direct (where higher values are better). The chart clearly shows that the proposed model (green line) outperforms the baselines, as its performance area fully encompasses both baselines in BPP, training cost, and computational cost, with only a negligible reduction in PSNR. This minor PSNR drawback is attributable to the high computational and training demands of the baseline pure-transformer approach.

### 4.4. Differentiable Learned Quantization

The proposed research introduces a differentiable learned quantization module that improves both compression efficiency and reconstruction quality. In a scenario where this module is not present the encoder outputs a continuous latent variable z, typically regularized to match a simple Gaussian prior N(0,I) via a Kullback-Leibler (KL) divergence term. Reconstructions produced by such models often appear blurry, smooth, or washed out, particularly in regions containing high-frequency content such as fine textures, sharp edges, or detailed structures. This behavior is largely due to the use of an $\ell_{2}$ (mean squared error) reconstruction loss, which is minimized by predicting the conditional mean of all possible pixel values at each spatial location. Averaging across multiple plausible sharp reconstructions inevitably leads to blurred outputs. The continuous nature of the latent space further allows the decoder to generate arbitrary combinations of pixel values, reinforcing this averaging effect.

**Figure 7:**
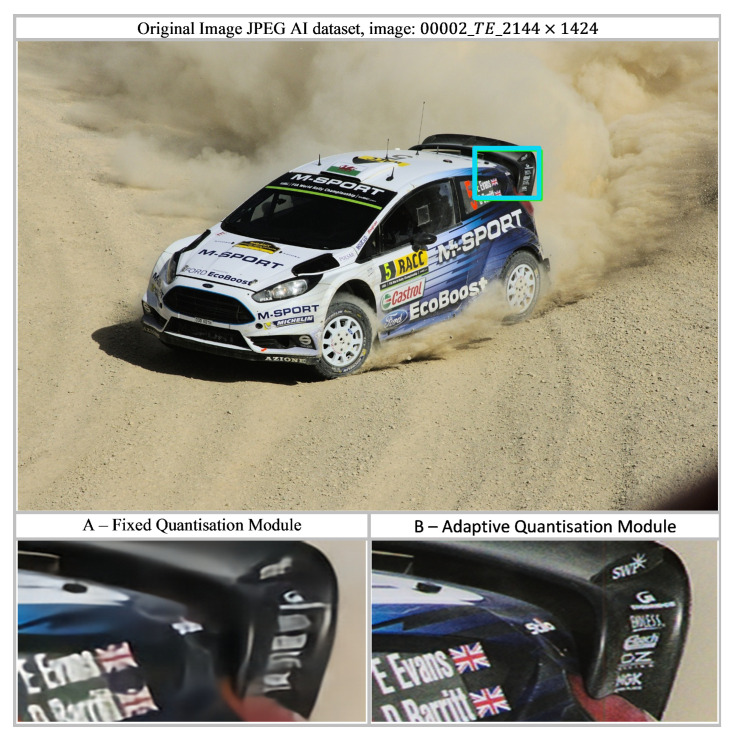
A region of interest (ROI) is extracted from the original image 00002_TE_2144×1424 from the JPEG AI dataset, which has dimensions of 2144×1424 pixels. (**A**) Fixed quantisation module; (**B**) Adaptive quantisation module. The ROI, indicated by a blue rectangle, is extracted consistently from the standard quantization method with fixed codebooks, and a learned quantization method with adaptive codebooks. (**A**) corresponds to standard quantization, while (**B**) corresponds to learned quantization. As illustrated in the figure, standard quantization leads to noticeable blurring and over-smoothing, whereas learned quantization preserves sharp text, clear edges, and well-defined line structures.

In contrast, the proposed approach employs a VAE with an intermediate learned quantization stage and a learned codebook. This design discretizes the latent space, forcing the encoder’s continuous outputs to be mapped to a finite set of learned codebook vectors, often referred to as visual tokens. The resulting discrete bottleneck prevents the decoder from producing averaged solutions. Instead, the decoder learns to reconstruct images as compositions of these discrete tokens, each corresponding to a specific visual pattern. As a result, the reconstructed images are significantly sharper and more faithful, preserving fine textures, crisp edges, and intricate structural details as observed in Figure 7.

### 4.5. Visual Analysis of Reconstruction Artifacts

The visual analysis of reconstructed images provides critical subjective insight into the superior performance of the proposed model, directly confirming the implications of the high objective metrics. Crucially, the Patch-Based Swin Transformer hyperprior mitigates the common artefacts associated with simpler entropy models while effectively retaining both image structure and perceptual quality.

**Figure 8:**
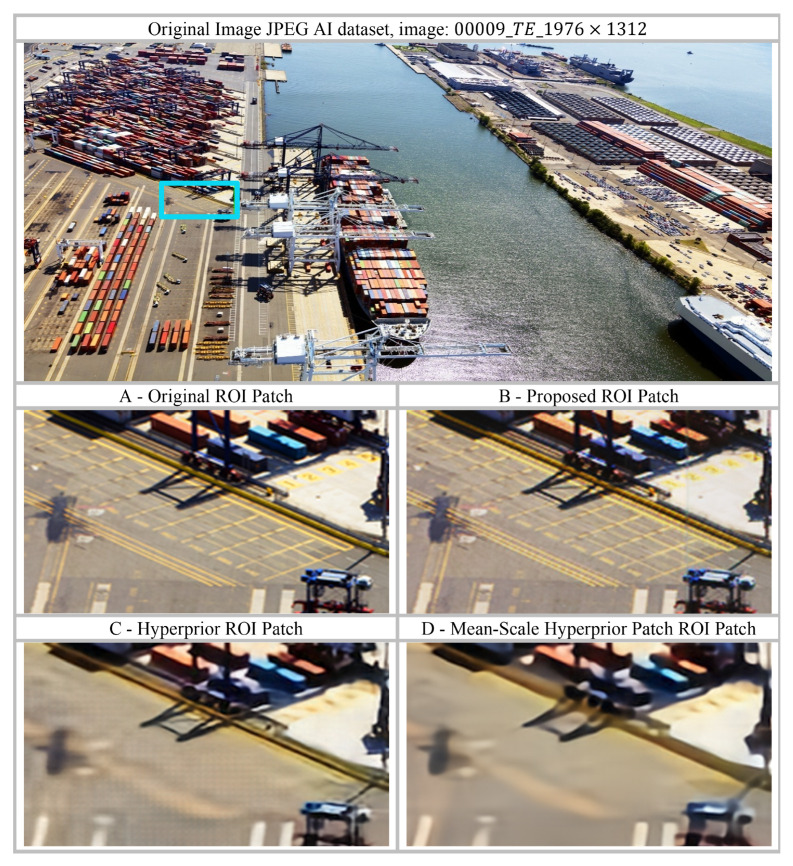
Visual analysis of reconstruction artifacts based on local and global feature preservation, comparing the proposed method with baseline Hyperprior and Mean-Scale Hyperprior models. (**A**) Original image patch; (**B**) Reconstructed patch from the proposed method; (**C**) Reconstructed patch from the baseline Hyperprior model; (**D**) Reconstructed patch from the Mean-Scale Hyperprior model. The visualization is based on image 00009_TE_1976×1312 from the JPEG AI dataset (dimensions: 1976×1312×3). The blue rectangle indicates the region from which patches were extracted.

The analysis of the Hyperprior and Mean-Scale Hyperprior models reveals a consistent failure in handling the complex spatial dependencies necessary for high-fidelity reconstruction, as illustrated in Figure 8. These models exhibit significant over-smoothing, where sharp edges such as the boundaries of shadows or the linear elements of the crane are washed out and softened. This collective flaw arises because these simpler models are unable to accurately capture the fine-grained correlations in the compressed data, forcing them to compromise between compression rate and detail preservation, resulting in visual artifacts and a substantial loss of the original image’s crispness.

**Figure 9:**
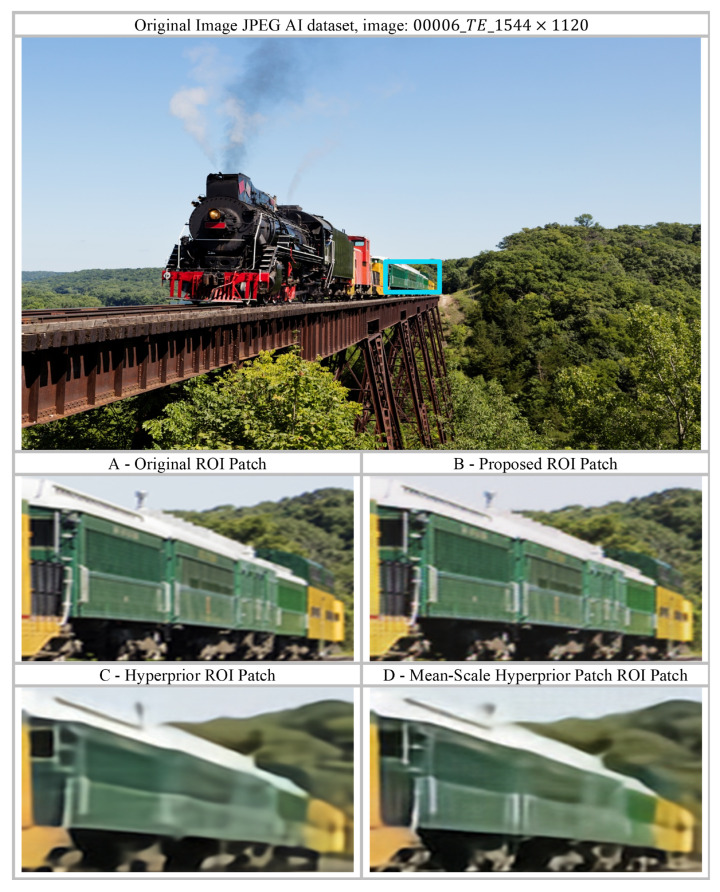
Visual analysis of reconstruction artifacts based on local and global feature preservation, comparing the proposed method with the baseline Hyperprior and Mean-Scale Hyperprior models. (**A**) Original image patch; (**B**) Reconstructed patch from the proposed method; (**C**) Reconstructed patch from the baseline Hyperprior model; (**D**) Reconstructed patch from the Mean-Scale Hyperprior model. The visualization is based on 00006_TE_1544×1120 from the JPEG AI dataset, with dimensions 1544×1120×3. The patches shown are extracted from the same region in the original image, the proposed model output, and the baseline models. The blue rectangle in the top image indicates the region from which patches A, B, C, and D were extracted. The patches have been enlarged to clearly illustrate and compare the performance of the different models.

A similar trend is observed in Figure 9 for another image from the JPEG AI dataset (00009_TE_1976×1312). In this example, the regions of interest (ROI) corresponding to (C) Hyperprior and (D) Mean-Scale Hyperprior exhibit noticeable blurring and over-smoothing, with edges, textures, and structural details largely suppressed. This behavior is attributed to inefficient entropy modelling, which limits the ability of the baseline methods to preserve high-frequency information during compression. In contrast, the proposed method retains fine-grained details, as evidenced by clearly defined edges, visible texture patterns, and a distinct boundary where the yellow section of the carriage begins. Additionally, in the forest region above the train, the baseline models tend to merge the train structure with the background vegetation, whereas the proposed approach maintains a clear separation between these distinct image components, demonstrating improved structural preservation.

In stark contrast, the proposed method utilizing the patch-based Swin Transformer hyperprior transcends these limitations and delivers results that are remarkably faithful to the original. This architectural leap is crucial: the Swin Transformer’s ability to model dependencies hierarchically and compute self-attention through shifted windows allows it to effectively integrate both local fine details and global structural coherence. Visually, this translates to the stunning preservation of lines and edges, where the fidelity of small vehicles, the rigid geometry of the docking area, and the sharpness of cast shadows are maintained perfectly. This high-fidelity performance confirms that the Swin Transformer provides a vastly more accurate entropy model for the latent features, allowing the algorithm to compress the image highly efficiently while meticulously retaining the necessary high-frequency information that defines visual quality.

Based on these observations, the visual data strongly substantiates the claim that the patch-based Swin Transformer hyperprior sets a new standard for learned image compression. By successfully overcoming the ubiquitous challenges of blurring and over-smoothing that characterize simpler models, the proposed architecture provides superior perceptual quality. It demonstrates that a powerful, attention-based prior is essential not only for achieving better compression ratios but, critically, for ensuring the precise preservation of structural integrity.

## 5. Conclusions

This work successfully addresses a critical limitation in existing Variational Autoencoder-based learned image compression models: the rigidity of simplistic entropy priors. We have introduced a novel framework that replaces these conventional priors with a Patch-Based Swin Transformer network, which acts as a sophisticated hyperprior, dynamically learning the complex, high-dimensional dependencies of the latent space. The experimental results definitively validate our approach, demonstrating superior Rate-Distortion performance across multiple standard quality metrics compared to the Factorized Prior, Hyperprior, and Mean-Scale Hyperprior baselines. Most notably, the model achieves exceptionally high MS-SSIM scores (∼0.998) at high compression rates. This commanding performance in the Multi-Scale Structural Similarity Index serves as direct, compelling evidence that the Swin Transformer’s windowed attention mechanism is highly effective at retaining both local (fine textures and details) and global (overall structure and long-range coherence) image features, which translates to a significantly enhanced perceptual quality in the reconstructed images.

Overall, by leveraging the representational power of modern transformer architectures to redefine the entropy model, our framework establishes a new paradigm for learned image compression. The substantial gains in compression efficiency and visual fidelity confirm that incorporating advanced neural network structures for context modelling is a powerful path forward. Future work, particularly the exploration of a Hierarchical Swin Prior, promises to further harness multi-scale contextual information to achieve even greater precision in bitrate estimation and, ultimately, push the boundaries of learned compression efficiency.

While the proposed framework achieves state-of-the-art Rate-Distortion performance, particularly in the preservation of local and global features, as evidenced by the high MS-SSIM scores, certain limitations remain that inform our future research directions, and these are as follows:

- Latent Feature Rigidity at Deep Scales: Although the Swin Transformer captures intricate dependencies, the overall latent space representation still adheres to the fixed hierarchical structure imposed by the autoencoder. The model’s ability to model extremely long-range, non-local dependencies across the entire image is still constrained by the windowed attention mechanism.
- Stitching Artifacts and Metric Degradation in Large Images: Due to the nature of computation for patch-based transformers, the final step of stitching the compressed patches back together for large images introduces a stitching effect or boundary artifacts. This causes a reduction in overall performance metrics (PSNR, SSIM, and MS-SSIM) compared to the higher scores observed within individual patches. The overlap regions are slightly less accurate, revealing a weakness that future research should address.

Our primary future direction is to incorporate a cross-attention mechanism within the latent space to enable efficient patch-to-patch communication. Operating in the latent domain is significantly less computationally expensive than applying attention at the VAE encoder level, while still allowing the model to capture long-range dependencies across spatial regions. By facilitating interaction between latent patches, this approach aims to enhance global context modelling, improve structural consistency, and further preserve fine details in the reconstructed images ultimately leading to improved compression performance and perceptual quality. In parallel, we aim to address stitching artifacts observed between adjacent patches by enhancing the training objective potentially through a loss function conditioned to emphasize patch edges and boundary consistency. By combining multi-scale feature extraction with edge-aware optimization, the model will not only better capture global and local correlations but also produce seamlessly stitched reconstructed frames with minimal discontinuities at patch boundaries.

## Figures and Tables

**Table 1:** Summary of models with their corresponding λ values and the number of convolution channels in each of the 3 layers of the encoder model.

Model	λ	Channels [Ch1, Ch2, Ch3]
1	0.1	[64, 128, 256]
2	0.5	[64, 128, 256]
3	1	[64, 128, 256]
4	10	[64, 128, 256]
5	100	[96, 192, 384]
6	250	[96, 192, 384]
7	750	[128, 256, 512]
8	1000	[128, 256, 512]

**Table 6:** Performance comparison of transformer-based models across three benchmark datasets, including compression metrics, computational requirements, and training costs.

Dataset	Model	BPP	PSNR (dB)	GPU Units	FP32 Perf.	Training (h)
Kodak	SwinT-Hyperprior	1.16941	37.77067	5120	∼15.7 TFLOPS	240
	SwinT-Charm	1.11675	37.79939	5120	∼15.7 TFLOPS	336
	Proposed	0.86215	36.99908	2560	∼8.1 TFLOPS	2
JPEG AI	SwinT-Hyperprior	1.16143	36.73541	5120	∼15.7 TFLOPS	240
	SwinT-Charm	1.10977	36.77166	5120	∼15.7 TFLOPS	336
	Proposed	1.08020	36.49340	2560	∼8.1 TFLOPS	2
CLIC	SwinT-Hyperprior	0.81555	39.16766	5120	∼15.7 TFLOPS	240
	SwinT-Charm	0.77897	39.22077	5120	∼15.7 TFLOPS	336
	Proposed	0.64040	36.78010	2560	∼8.1 TFLOPS	2

**Table 7:** Bitrate overhead comparison: BPP increase of transformer-based baselines relative to the proposed method across three benchmark datasets.

Dataset	Benchmark Model (BPP)	BPP Increase vs. Proposed
Kodak	SwinT-Hyperprior (1.169 BPP)	35.64%
	SwinT-Charm (1.117 BPP)	29.52%
JPEG AI	SwinT-Hyperprior (1.161 BPP)	7.51%
	SwinT-Charm (1.110 BPP)	2.74%
CLIC	SwinT-Hyperprior (0.816 BPP)	27.39%
	SwinT-Charm (0.779 BPP)	21.64%

**Table 8:** PSNR performance comparison: Quality deficit and percentage dominance of transformer-based baselines over the proposed method across three benchmark datasets.

Dataset	Model (PSNR)	PSNR Deficit vs. Proposed	PSNR Dominance (%)
Kodak	SwinT-Charm (37.80 dB)	−0.80 dB	2.16%
	SwinT-Hyperprior (37.77 dB)	−0.77 dB	2.08%
JPEG AI	SwinT-Charm (36.77 dB)	−0.28 dB	0.76%
	SwinT-Hyperprior (36.74 dB)	−0.25 dB	0.68%
CLIC	SwinT-Charm (39.22 dB)	−2.44 dB	6.64%
	SwinT-Hyperprior (39.17 dB)	−2.39 dB	6.50%

## Data Availability

The original data presented in the study are openly available at https://jpegai.github.io/test_images/ (accessed on 3 March 2025); https://github.com/MohamedBakrAli/Kodak-Lossless-True-Color-Image-Suite/tree/master (accessed on 3 March 2025); http://data.vision.ee.ethz.ch/cvl/DIV2K/DIV2K_train_HR.zip (accessed on 3 March 2025); http://data.vision.ee.ethz.ch/cvl/DIV2K/DIV2K_valid_HR.zip (accessed on 3 March 2025); and https://clic2025.compression.cc/ (accessed on 10 December 2025).

## Abbreviations

The following abbreviations are used in this manuscript:

BPP	Bits per pixel
PSNR	Peak Signal-to-Noise Ratio
SSIM	Structural Similarity Index Measure
MS-SSIM	Multi-Scale Structural Similarity
